# Potential Mediators of a School-Based Digital Intervention Targeting Six Lifestyle Risk Behaviours in a Cluster Randomised Controlled Trial of Australian Adolescents

**DOI:** 10.1007/s11121-023-01616-z

**Published:** 2023-12-20

**Authors:** Siobhan M. O’Dean, Matthew Sunderland, Scarlett Smout, Tim Slade, Cath Chapman, Lauren A. Gardner, Louise Thornton, Nicola C. Newton, Maree Teesson, Katrina E. Champion

**Affiliations:** 1 The Matilda Centre for Research in Mental Health and Substance Use, The University of Sydney, Darlington Campus, Level 6, Jane Foss Russell Building, Sydney, NSW Australia; 2 School of Medicine and Public Health, The University of Newcastle, Callaghan, Australia

**Keywords:** Risky lifestyle behaviours, Prevention, Multiple health behaviour change, Mediation

## Abstract

Lifestyle risk behaviours—physical inactivity, poor diet, poor sleep, recreational screen time, and alcohol and tobacco use—collectively known as the “Big 6” emerge during adolescence and significantly contribute to chronic disease development into adulthood. To address this issue, the Health4Life program targeted the Big 6 risk behaviours simultaneously via a co-designed eHealth school-based multiple health behaviour change (MHBC) intervention. This study used multiple causal mediation analysis to investigate some potential mediators of Health4Life’s effects on the Big 6 primary outcomes from a cluster randomised controlled trial of Health4Life among Australian school children. Mediators of knowledge, behavioural intentions, self-efficacy, and self-control were assessed. The results revealed a complex pattern of mediation effects across different outcomes. Whilst there was a direct effect of the intervention on reducing moderate-to-vigorous physical activity risk, the impact on sleep duration appeared to occur indirectly through the hypothesised mediators. Conversely, for alcohol and tobacco use, both direct and indirect effects were observed in opposite directions cancelling out the total effect (competitive partial mediation). The intervention’s effects on alcohol and tobacco use highlighted complexities, suggesting the involvement of additional undetected mediators. However, little evidence supported mediation for screen time and sugar-sweetened beverage intake risk. These findings emphasise the need for tailored approaches when addressing different risk behaviours and designing effective interventions to target multiple health risk behaviours. The trial was pre-registered with the Australian and New Zealand Clinical Trials Registry: ACTRN12619000431123.

**Supplementary Information:**

The online version contains supplementary material available at 10.1007/s11121-023-01616-z.

## Introduction

Lifestyle risk behaviours, including physical inactivity, poor diet, poor sleep, recreational screen time, and alcohol and tobacco use, collectively known as the “Big 6” are significant contributors to chronic disease development, including type 2 diabetes, cancer, cardiovascular disease, obesity, and mental health disorders (Murray et al., 2020). These risk factors commonly emerge during adolescence and persist into adulthood. Multiple health behaviour change (MHBC) interventions targeting these behaviours simultaneously may offer a cost-effective and efficient approach to promoting healthy lifestyles in adolescence (Prochaska et al., 2008). However, few eHealth MHBC interventions have been rigorously tested, none have targeted all of the Big 6, and effects have been small and short-term. To address these gaps, Health4Life was co-designed with adolescents, educators, and health experts as the first eHealth school-based MHBC intervention to target the Big 6 (Champion et al., 2020).

Previous analyses from a cluster randomised controlled trial of Health4Life found little evidence for greater efficacy of the intervention in modifying the Big 6 risk behaviours compared to health education as usual (active control) (Champion et al., 2023). However, analyses of the effects of Health4Life on knowledge of health behaviours are promising. Specifically, participants who received the Health4Life intervention reported improved knowledge about the Big 6 risk behaviours over 24 months (Champion et al., 2023). Potential reasons for the limited evidence for intervention efficacy on behaviour change are detailed in Champion et al. (2023). Briefly, although the intervention significantly increased knowledge of the risky behaviours, the context of a global pandemic in which participants experienced ongoing disruptions to their lifestyles over 2 years could have reduced opportunities for students to enact knowledge and positive intentions for change gained through the program. Moreover, although knowledge gains are crucial in explaining why lifestyle changes are important, they alone are generally insufficient for behaviour change (Arlinghaus & Johnston, 2018).

Effective implementation and translation of health behaviour interventions relies on understanding *why* people change health behaviours (mediators) and *what* behaviours they can change (outcomes) (Rothman & Sheeran, 2020). Previously, researchers have advocated for the importance of examining mediators in the absence of an intervention effect to further understand how and why such effects may have failed to emerge. O’Rourke and MacKinnon (2018) explain several potential conditions that could account for a significant mediation effect, even in the absence of an intervention effect, including (1) when the mediated effect and the total effect are equal and the mediated effect provides more statistical power to detect effects, (2) inconsistent/competitive mediation (the mediated and direct effects are in opposing directions, (3) increased power to detect mediation with multiple mediators, and (4) multiple mediators with effects in opposing directions. As such, having a deeper understanding of the mechanisms or mediators through which interventions like Health4Life may or may not improve health behaviours is integral to understanding, improving, and implementing future MHBC interventions.

Multiple systematic reviews have investigated and summarised the findings on potential mediators of health behaviour change interventions in adolescents (Cerin et al., 2009; Kelly et al., 2017; Lubans et al., 2008; Van Stralen et al., 2011). Self-efficacy—the belief in one’s own ability or capacity to engage in behaviours to achieve goals—as well as knowledge and positive and negative attitudes towards target behaviours were commonly investigated as mediators of health behaviour change interventions. The first review synthesised seven studies focused on mediators of physical activity among secondary school-age students, including self-efficacy to change behaviour, attitudes, and interpersonal factors. Self-efficacy was found to be a strong mediator of physical activity (Lubans et al., 2008). Cerin et al.’s (2009) review evaluated mediators of dietary change across seven studies, finding that increased self-efficacy and positive outcome expectations were most consistently associated with dietary behaviour. Van Stralen et al.’s (2011) review found evidence supporting self-efficacy and intentions as mediators of physical activity, whilst improved attitudes, knowledge, and habit strength were mediators of dietary behaviour interventions. A more recent review found that autonomous motivation (the intrinsic drive to pursue activities based on personal values or enjoyment) was the only consistent significant mediator for the effects of behaviour change interventions on reducing screen time (Kelly et al., 2017). Consistent with other reviews, the authors also reported that self-efficacy and goal intentions were significant mediators of dietary interventions. In contrast to earlier reviews, self-efficacy was not a consistent direct mediator of physical activity interventions, but goal intentions and perceived barriers to change were significant mediators (Kelly et al., 2017). That is, interventions that were able to improve intentions to fulfill, belief in capacity to reach goals, and reduce perceived barriers were more likely to result in dietary and activity behaviour change.

Studies have also investigated mediators of school-based substance use prevention programs for adolescents; however, findings are somewhat inconsistent. For instance, one study reported that increasing anti-alcohol attitudes, refusal assertiveness, decreasing risk taking, reducing intentions to use, and reinforcing peer normative expectations were important mediators of efficacy for an alcohol prevention program (Botvin & Griffin, 2015). In contrast, two studies found that only decreasing positive beliefs about the consequences of substance use mediated the effects of an alcohol intervention (Longshore et al., 2007) and an alcohol and tobacco use program (Orlando et al., 2005) on subsequent alcohol use. Other studies have found a combination of these mediators (increasing refusal skills, knowledge, and self-efficacy and decreasing positive attitudes, normative perceptions of peer use, and reasons to use) were significant predictors of tobacco use prevention programs (Giannotta et al., 2014; Harrell Stigler et al., 2011). Findings from these studies in comparison to those from other health behaviour change interventions suggest the underlying mechanisms for prevention of the Big 6 might vary between behaviours. That is, for example, the factors needing to be targeted for changes to increase physical activity (e.g., goal-intentions, self-efficacy) may be different from those needing to prevent the uptake of alcohol and tobacco use (e.g., refusal skills, normative belief change) and reducing screen time (e.g., autonomous motivation). Understanding how different mechanisms might mediate different aspects of MHBC interventions is crucial for designing programs that can effectively and efficiently address the different factors associated with changing each type of behaviour.

The Health4Life program is based on a MHBC approach that integrates theories of social influence (Botvin, 2000), social cognition (Bandura, 1986), self-determination (Ryan & Deci, 2000), and the two-process model of sleep (Borbely, 1982) to address the Big 6 risk behaviours. The program uses behaviour change techniques, such as providing evidence-based information, enhancing resistance skills, modifying normative beliefs, developing life skills, self-regulatory skills, and promoting autonomy, relatedness, and competence. These techniques are integrated into co-designed cartoon-based modules designed to improve skills in decision-making, problem-solving, coping, self-control, goal setting, and self-monitoring. The program aims to increase autonomous motivation by providing students with the skills and knowledge they need to enact behaviour change (see the Health4Life conceptual model in Champion et al., 2020). Of these hypothesised mechanisms, the cluster randomised controlled trial (cRCT) collected data on knowledge (including normative beliefs), general self-efficacy, behavioural intentions to change, and self-control. This study examines if these mechanisms mediate the intervention’s effect on the six primary outcomes: alcohol and tobacco use, moderate-to-vigorous physical activity, recreational screen time, sleep duration, and sugar-sweetened beverage consumption.

## Method

### Design

**Fig. 1:**
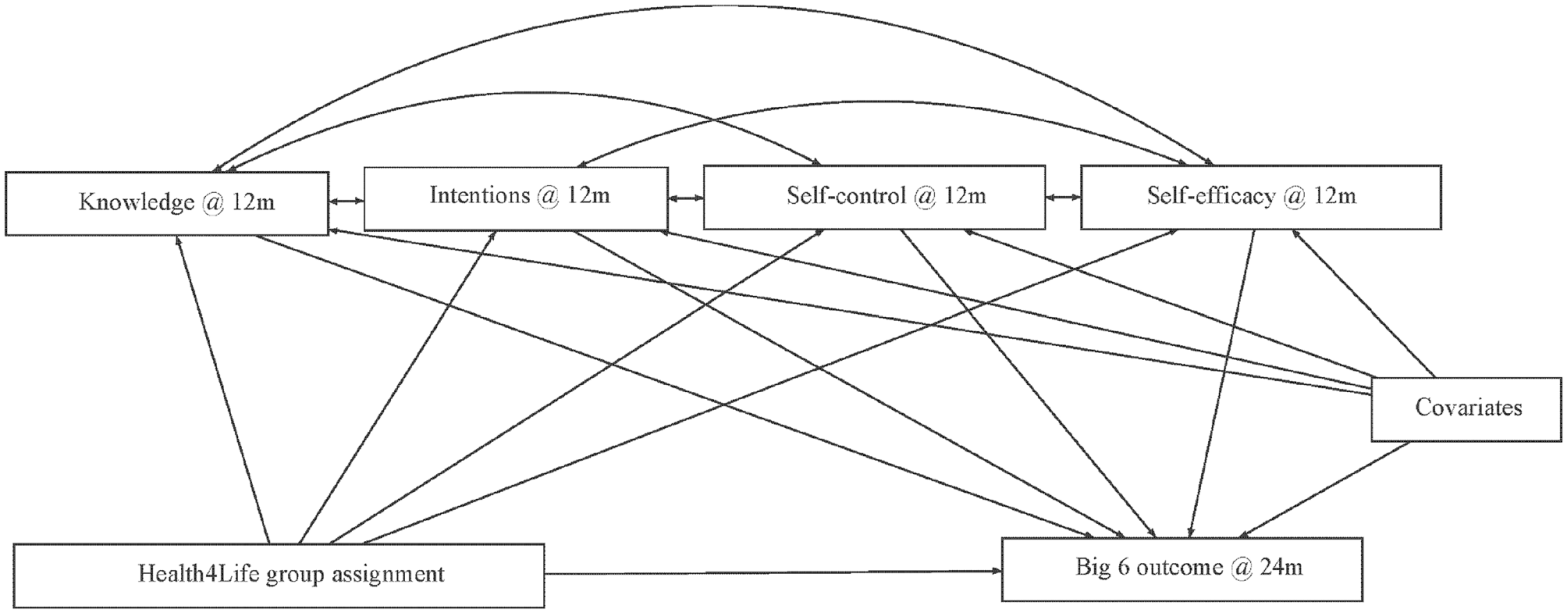
Path diagram for causal multiple mediation model of Health4Life on Big 6 outcomes. Note: Separate models were estimated for each Big 6 outcome

Data for this study were derived from a cRCT of the Health4Life intervention. A total of 71 schools participated in the study, for which baseline measurements were taken in 2019. Stratified block randomisation assigned schools to one of two conditions: (1) Health4Life, or (2) active control (health education as usual). Randomisation was stratified by school site (New South Wales metro, New South Wales regional, Queensland, or Western Australia) and school sex composition (co-educational, predominantly male (> 60%) or predominantly female (> 60%)). The Consolidated Standard of Reporting Trials (CONSORT) diagram summarises participant recruitment and retention rates for both the intervention and control groups in Fig. 1. The study protocol, including sample size calculations and consent procedures was approved by Human Research Ethics Committees of the University of Sydney (2018/882), the University of Queensland (2019000037), Curtin University (HRE2019–0083), and relevant school sector ethics committees. Full details of the study protocol are reported elsewhere (Teesson et al., 2020).

### Participants

All year 7 students who attended participating schools in 2019 and were fluent in English were eligible to participate. Consent procedures varied among schools, with some using opt-out parental consent and others requiring written or oral opt-in consent. 6639 students completed the baseline questionnaire. All students were invited to participate in self-reported follow-up surveys immediately post-intervention (~ 7 weeks), as well as 12 months (2020), 24 months (2021), and 36 months (2022) after baseline assessment. Study retention was high, with 97% of participants providing follow-up data on at least one occasion (*N* = 6454) and 86% providing data on two or more assessments (*N* = 5698). In terms of reported sex at birth, the total sample comprised approximately 50% male, 49% female, and 1% preferred not to say. The average age of the sample was 13 years at baseline and 15 years at 24-month follow-up (when the primary outcomes were assessed).

### Interventions

Schools randomised to the Health4Life condition were asked to administer the Health4Life intervention during health education classes. The Health4Life intervention used a staged model of prevention, including both universal and selective programs. The universal intervention comprised of 6 online cartoon modules that used co-designed storylines to convey evidence-based information about the Big 6. These cartoons were delivered sequentially in regular health and physical education lessons. Students also received targeted, web-based feedback after each questionnaire, comparing their reported behaviours to national health guidelines. The selective component of the intervention was available to those who reported behaviours at risky levels for 2 or more of the Big 6 and included additional cognitive behavioural and motivation-enhancement techniques delivered through a companion smartphone app. The Health4Life intervention drew on multiple behavioural theories, including self-determination theory, which describes a behaviour change pathway from “amotivation” to “motivation”. Self-determination theory emphasises that increasing the perceived value of a behaviour is essential for influencing intentions (Ryan & Deci, 2000; Teesson et al., 2020). Health4Life used social influence theory to enhance perceived value through co-designed cartoon story-based lessons featuring characters the same age as students, educating them about the Big 6, and targeting normative perceptions simultaneously (Champion et al., 2020). More detail on specifics of the intervention can be found in Champion et al. (2020), Teesson et al. (2020), and Thornton et al. (2021). Schools in the active control group followed their regular health education curriculum, with teachers recording any education related to Big 6 in a logbook. Out of the 35 control schools, 32 provided logbook data from 96 teachers, and most teachers (90 out of 96) reported teaching at least one of the Big 6 health education lessons in 2019 (Champion et al., 2023).

### Measures

#### Outcomes

##### Substance Use

Alcohol use was assessed with a single item and a standard drink pictorial chart: “Have you had a full standard alcoholic drink in the past 6 months?” (0 = no, 1 = yes). Tobacco use was measured with a single item: “In the past 6 months, have you tried cigarette smoking, even one or two puffs?” (0 = no, 1 = yes).

##### Screen Time

Screen time was assessed with mean time (minutes and hours) spent engaged in recreational screen time on weekends and weekdays in the past 7 days. The derived screen time variable for the analysis was binary and indicated whether participants met screen time guidelines (< 2 h per day) or not (0 = meets guidelines, 1 = does not meet guidelines).

##### Moderate-Vigorous Physical Activity (MVPA) Risk

MVPA was measured with a single item that assessed the number of days in the past 7 days participants had engaged in at least 60 min of MVPA. The derived MVPA variable was coded as binary and indicated whether participants met guidelines (7 days) or not (0 = meets guidelines, 1 = does not meet guidelines).

##### Sugar Sweetened Beverages (SSB) Risk

A single item assessed the typical consumption of sports drinks, cordials, or soft drinks. The derived SSB variable was binary and indicated whether participants drank 2 or more cups of SSBs per week (0 = 1 or less cups a week, 1 = 2 or more cups a week).

##### Sleep Risk

Mean sleep duration (minutes and hours) was calculated with a 6-item scale (Champion et al., 2023), which was then used to derive a binary variable indicating whether participants met sleep guidelines (8–10 h per night) or not (0 = meets guidelines, 1 = does not meet guidelines).

#### Mediators

##### Knowledge

Knowledge was measured using a 20-item scale that was designed to reflect the content of Health4Life. The scale included questions about Australian health guidelines for the Big 6, the prevalence of alcohol and tobacco use among Australian adolescents, and the physical and mental health effects of the Big 6. The total knowledge score was obtained by summing the scores for all items on the scale. The details of the scale can be found in Champion et al. (2023).

##### General Self-Efficacy

Participants completed the General Self-Efficacy Scale (Schwarzer & Jerusalem, 1995), a self-report questionnaire that measured their self-efficacy at each time point. Ten items (e.g., “I can usually handle whatever comes my way”) were measured on a 4-point scale ranging from 1 (not at all true) to 4 (exactly true). Total scores could range from 10 to 40.

##### Self-Control

Self-control was measured using the 13 items Brief Self-control Scale (Tangey et al., 2004) adapted for adolescents. Total scores ranged from 14 to 65 at baseline.

##### Behavioural Intentions

Participants responded to 6 items assessing their intentions to engage in or change their Big 6 health behaviours. All behavioural intention variables were operationalised as ordinal scales. For alcohol and tobacco use*,* participants were asked how likely it was they would try alcohol and cigarettes (tobacco) in the future and were measured on a scale of 0 (very unlikely) to 4 (very likely). For MVPA, screen time, sleep, and SSB, participants were asked to indicate the extent to which—over the next 3 months—they intended to be physically active on all or most days of the week and reduce their screen time on all or most days of the week, sleep for 9–11 h per night on all or most days of the week, and finally swap energy drinks, soft drinks, sports drinks, or cordial for water on all or most days of the week. Reponses were on a 4-point scale ranging from 0 (not at all true of me) to 3 (very true of me).

### Statistical Analysis

We conducted causal multiple mediation analysis using structural equation modelling (as detailed in Fig. 1). Each model was estimated using a weighted least squares mean and variance adjusted (WLSMV) estimator with a probit link (Nguyen et al., 2016). Separate models investigated the effects associated with each of the Big 6 primary outcomes at the 24-month follow-up time point (as previously determined as the primary endpoint in the Health4Life protocol). All mediators were measured at the 12-month follow-up time point. The exposure variable was randomised group assignment to either Health4Life or control group at baseline. All analyses adjusted the standard errors for the non-independence of observations due to clustering of students nested within schools, as is typical for cRCTs, using a robust sandwich estimator. Similarly, additional covariates in the models included the randomisation stratification variables (sex, school location), age, baseline psychological distress given potential confounding on outcomes and mediators (measured using the Kessler 6 psychological distress scale; Kessler et al., 2002), and the mediators and outcomes measured at baseline. Multiple imputation, using five imputed datasets, was used to account for missing data in the outcomes, mediators, and covariates, in a Bayesian framework under a missing at random assumption. All analyses were completed in Mplus version 8 and R/RStudio (version 4.2.3) using the MplusAutomation package (Hallquist & Wiley, 2018).

All mediators were included in single models for each outcome to determine the combined mediation effects. For behavioural intentions, the relevant intention item was included for each outcome, e.g., intention to meet sleep recommendations for sleep risk and intentions to engage in MVPA for MVPA risk. The combined effects of the multiple mediators, in addition to the total effect, were calculated on the risk difference scale (RD) and risk ratio scale (RR) using the method and adapted code outlined in Nguyen et al. (2016). Risk difference and risk ratios are standard effect sizes for binary outcomes and reflect the difference in the prevalence of the outcome depending on the administration of the intervention. Standard errors and 95% confidence intervals were estimated in 500 bootstrap samples.

The current study uses a causal mediation analysis approach, which differs from traditional mediation models by including the interaction between the exposure (intervention group) and mediator variables (Rijnhart et al., 2021). This interaction creates a set of four probabilities for each outcome under hypothetical assumptions reflecting different potential outcomes and counterfactuals. These probabilities (P) are defined in the current study as follows: (1) the probability of the outcomes at 24 months under the hypothetical situation where all participants are assigned to the Health4Life group, and the mediators are set at the values observed in the Health4Life group (P11; i.e., observed probability), (2) the probability of the outcomes at 24 months when all participants are assigned to the Health4Life group, and the mediators are set at the values observed in the control group (P10; i.e., counterfactual probability), (3) the probability of the outcomes at 24 months when all participants are assigned to the control group, and the mediators are set at the values observed in the Health4Life group (P01; i.e., counterfactual probability), and (4) the probability of the outcomes at 24 months when all participants are assigned to the control group and the mediators are set at the values observed in the control group (P00; observed probability).

These four probabilities are then used to estimate direct and indirect effects by finding the difference between different combinations of observed probabilities and counterfactual probabilities depending on whether the mediator values are held constant or the intervention values held constant. In the current study, the pure natural direct effect (PNDE) and the total natural indirect effect (TNIE) were estimated. The PNDE represents the direct effect of changing each participant’s exposure value (i.e., comparison between the Health4Life and control groups) on the outcome under the hypothetical condition that the mediator values for all participants are held constant at the control group’s observed level (e.g., PNDE = P10−P00). The TNIE represents the indirect intervention effect on the outcome via the mediators, under the hypothetical condition that the Health4Life intervention remains constant, and the mediators change from the values that are naturally observed in the Health4Life group to the values observed in the control group (e.g., TNIE = P11−P10). Finally, the total effect (TE) is calculated as the intervention’s total effect on the outcome, which includes the sum of both the direct (PNDE) and indirect effects (e.g., TE = PNDE + TNIE).

For the results to be considered causal, several strong assumptions need to be met. These include (1) the absence of confounding between intervention and mediators, (2) the absence of confounding between intervention and outcome, (3) the absence of confounding between mediators and outcome, and (4) the absence of mediator-mediator interactions that influence the outcome, in the case of multiple mediators. Randomised assignment of participants to intervention and control conditions can help address the first two assumptions, whereas sensitivity analysis and controlling for additional known covariates can help address the second two assumptions.

The sensitivity analysis proposed by Imai et al. (2010) for causal mediation with more than two mediators is currently not available (Nguyen et al., 2016). Despite inclusion of several covariates, there remains the possibility that unmeasured confounders may fully explain the observed associations. To test the robustness of the findings and the validity of the assumptions, mediation E-values were estimated. E-values represent the required strength of an association between an unmeasured third variable (confounder) with the intervention and mediators as well as with the intervention and outcomes that would render the identified associations statistically insignificant (Smith & VanderWeele, 2019). Mediation E-values are estimated as rate ratios, and values near 1.00 indicate less confidence that the results are robust against unmeasured confounders.

## Results

The results of the causal mediation analysis for all outcomes are provided in Table 1 (with coefficients associated with the full models for each outcome as described in Fig. 1 provided in the Supplementary material). Across the four mediators and six outcomes, the intervention was consistently associated with increased knowledge, whereas increased intentions were associated with the sleep risk model. The intervention was not associated with increased self-control nor self-efficacy in any of the models. The total effects for all outcomes on the risk difference scale ranged from a 3.0 percentage point reduction in MVPA risk for the intervention group to a 1.8 percentage point increase in SSB risk in the intervention group. Bootstrap confidence intervals suggested that the total effect of the intervention on MVPA at 24-month follow-up was significantly different from zero, whereas there was little evidence for a significant total effect of the intervention on the five other risk behaviours. Of the total effect of the intervention on MVPA, the direct and indirect effects suggest that this effect occurs directly from the intervention to the outcome rather than indirectly via the mediators. Under the hypothetical condition that the whole sample received the Health4Life intervention, but the mediators were held at levels naturally observed in the control group, the percentage of students reporting insufficient MVPA decreased by 3.4 percentage points (95% CI = −6.3,−1.5) at 24 months.

**Table 1:** Causal mediation effects of the H4L intervention on the Big 6 lifestyle risk behaviours

	**Total effect**						**Pure natural direct effect**						**Total natural indirect effect**					
	**RD**	**95% CI**		**RR**	**95% CI**		**RD**	**95% CI**		**RR**	**95% CI**		**RD**	**95% CI**		**RR**	**95% CI**	
**Alcohol use**	1.2%	−0.4%	2.9%	1.08	0.97	1.20	2.4%	0.6%	4.4%	1.15	1.04	1.30	−1.1%	−2.1%	−0.6%	0.94	0.89	0.97
**MVPA**	−3.0%	−4.9%	−1.1%	0.96	0.94	0.99	−3.4%	−6.3%	−1.5%	0.96	0.92	0.98	0.5%	−0.1%	2.7%	1.01	1.00	1.04
**Screen time**	−1.1%	−2.3%	0.1%	0.99	0.98	1.00	−0.7%	−2.6%	1.3%	0.99	0.97	1.01	−0.4%	−2.1%	1.2%	1.00	0.98	1.01
**Sleep risk**	−2.0%	−4.2%	0.2%	0.95	0.89	1.01	0.1%	−3.6%	5.3%	1.00	0.91	1.14	−2.1%	−6.8%	−0.3%	0.95	0.85	0.99
**SSB intake**	1.8%	−0.3%	3.8%	1.07	0.99	1.15	2.3%	−1.3%	8.0%	1.09	0.95	1.31	−0.5%	−5.9%	2.3%	0.98	0.83	1.10
**Tobacco use**	1.1%	0.0%	2.2%	1.19	1.00	1.43	1.8%	0.7%	3.8%	1.31	1.11	1.68	−0.7%	−2.2%	−0.2%	0.91	0.76	0.97

*MVPA* moderate-vigorous physical activity, *SSB* sugar-sweetened beverages, *RD* risk difference, *RR* risk ratio, *95% CI* 95% confidence intervals. Multiple mediators include knowledge, intentions, self-efficacy, and self-control. Models include age, sex, psychological distress, location, mediators at baseline, and outcomes measured at baseline as covariates

For alcohol use and tobacco use, despite there being an absence of a significant total effect, the mediation analysis indicated significant natural direct and indirect effects. Examining indirect effects, the impact of the change in the mediators on the outcomes (i.e., change from values naturally observed in the Health4Life group and the control group) under the condition that the whole sample were assigned to the Health4Life group, the percentage of students using alcohol and tobacco at 24-month follow-up is decreased by 1.1 (95% CI = −2.1,−0.6) and 0.7 percentage points (95% CI = −2.2,−0.2), respectively. However, examining direct effects, the impact of the change on the intervention group on the outcomes (i.e., change on outcomes between control and Health4Life groups) under the condition that the mediators were held at levels naturally observed in the control group, the percentage of students using alcohol and tobacco at 24-month follow-up increased by 2.4 (95% CI = 0.6, 4.4) and 1.8 percentage points (95% CI = 0.7, 3.8), respectively. That is, the indirect and direct effects may cancel each other out, resulting in a null observed total effect (a partial competitive mediation).

Finally, for those who failed to achieve the recommended guidelines for sleep, the total effect of the Health4Llife intervention was almost all accounted for by the TNIE, e.g., under the hypothetical condition that the whole sample received the H4L intervention, and the mediators change from values naturally observed in the intervention group relative to the control group, the number of students failing to meet sleep guidelines decreased by 2.1 percentage points (95% CI = −6.8,−0.3). Further inspection of the parameters estimated from the multiple mediation model (presented in the supplementary material) provides evidence that this indirect effect is primarily driven by an increased intention to improve sleep duration at the 12-month follow-up. This finding occurred despite the lack of evidence for a significant total effect and highlights the potential for increased power to detect effects via multiple mediators.

If the strong assumptions of the causal mediation analysis hold, these effects could be seen as causal. However, the results of the sensitivity analysis (Table 2) indicate that these findings might be at risk of unmeasured confounding given the small effect sizes and relatively small E-values. For example, the largest E-value was associated with the direct effect on tobacco use, indicating that an unmeasured confounder associated with both the mediators and the outcome with risk ratios of at least 1.97 would be sufficient to completely explain away the observed direct effect. From the remaining significant direct or indirect effects, the E-values ranged from 1.29 (for indirect effect on sleep) to 1.57 (for the direct effect on alcohol use). This means that a potential confounder would not have to be very strongly related to both mediators and the outcome to explain the observed findings.

**Table 2:** E-values associated with the mediation effects and the E-value for the 95% confidence interval

	**Pure natural direct effect E-values**		**Total natural indirect effect E-values**	
	**E-value**	**95% CI**	**E-value**	**95% CI**
**Alcohol use**	1.57	1.24	1.32	1.21
**MVPA**	1.25	1.16	1.11	1.00
**Screen time**	1.11	1.00	1.00	1.00
**Sleep risk**	1.00	1.00	1.29	1.11
**Sugar-sweetened beverage intake**	1.40	1.00	1.16	1.00
**Tobacco use**	1.95	1.46	1.43	1.21

E-values are in the risk ratio scale. The E-value can be interpreted as the risk ratio required between an unmeasured confounder and both the mediators and outcomes to completely explain away the observed effects. The E-value associated with 95% CI is interpreted as the risk ratio required between an unmeasured confounder with both mediators and outcome to shift the confidence interval to the null

## Discussion

The current study sought to identify potential mediating effects of knowledge, behavioural intentions, self-efficacy, and self-control when evaluating the impact of an eHealth school-based MHBC intervention on six lifestyle risk behaviours. Overall, the mediation effects differed depending on the different outcomes. There was a significant effect of the intervention on reducing MVPA risk, which appears to occur directly and bypass the hypothesised mediators (i.e., no mediation). Whereas, the significant effect of the intervention on sleep duration appears to occur indirectly via changes in the hypothesised mediators (i.e., full mediation). For alcohol and tobacco use, there appears to be both direct and indirect effects in opposite directions (i.e., competitive partial mediation), suggesting that the intervention not only reduced alcohol and tobacco use via the mediators but also increased alcohol and tobacco use independent of the hypothesised mediators, therefore cancelling out any total effect. For screen time risk and SSB intake risk, there was little evidence to suggest any mediation or total effects.

The results provide evidence for and against the hypothesised mediators with respect to MHBC outcomes and provides some indication of why the intervention might (and might not have) resulted in significant preventive effects in relation to the Big 6. For sleep risk, our results indicate that the intervention effect is indirectly related via change on the four mediators included in the analysis. Thus, for sleep at least, it appears there is some transition between improving intentions and improving behaviour for a small percentage of the sample. This finding is consistent with theories of behaviour change that argue that an individual must have an intention to change their behaviour for behaviour change to occur (e.g., the Theory of Planned Behavior (TPB), the Trans-Theoretical Model, and Self-Determination Theory; Ryan & Deci, 2000; Taylor et al., 2007).

For MVPA risk, there appears to be no mediation effect. These findings contrast past research that indicates intentions and self-efficacy are consistent and significant mediators of interventions designed to increase physical activity in adolescent populations (Lubans et al., 2008; Van Stralen et al., 2011). Reasons for the discrepancy in findings could be that these past studies focussed their interventions on physical activity alone (or physical activity and diet), whereas the Health4Life intervention included education on six health behaviours. As such, the Health4Life intervention may not have had adequate time devoted to bolstering self-efficacy and intentions around physical activity specifically. Moreover, according to Zhao and colleagues’ (2010) decision tree regarding the interpretation of different mediation conditions, these results provide some indication of an undetected mediator driving the observed intervention effect. This study does not provide an indication of what these undetected mediator(s) could be, and further hypothesis generation associated with MVPA is required. For screen time risk and SSB risk, there was little evidence that the intervention led to any significant direct or indirect improvement at 24-month follow-up over and above education as usual.

The results associated with alcohol and tobacco use provide a more complicated picture regarding the intervention and hypothesised mediators. Evidence of partial mediation was observed indicating that the intervention effect may be accounted for by both the hypothesised mediators as well as additional undetected mediators. The estimated direct effect of the intervention led to an increase in the total prevalence of past 6-month alcohol and tobacco use of 2.4 and 1.8 percentage points, respectively, a relatively small effect size with risk ratios of 1.15 and 1.31. A key question remains as to what the mechanisms potentially increasing prevalence of alcohol and tobacco use in the Health4Life group might be and whether these effects were limited to a specific subgroup of the total sample.

The wider social and environmental context that could have been influenced by the Health4Life intervention may provide one potential hypothesis. For example, increased open discussions related to alcohol and tobacco use in class time, at home, and among social groups in those who received the Health4Life intervention may have primed a small percentage of students to increase their substance use at follow-up in comparison to the control. Indeed, a previous review has shown that increases in the frequency of alcohol-specific communication between parents and young adolescents can lead to increases in alcohol use later in adolescence, particularly for poor-quality and inconsistent conversations discussing rules, consequences of use, and more permissive messages. However, high parental-child connectedness and good general communication and clear rule-setting have been shown to be protective (Carver et al., 2017). Thus, it may be important for future MHBC interventions to measure and monitor the frequency and quality of conversations and communication around alcohol and tobacco use among adolescents, parents, teachers, and their peers (McKay, 2015). Alternatively, it could be hypothesised that the self-report nature of these increasingly de-normalised outcomes could lead to such an increase in the intervention group. For example, the baseline estimates may have been subject to self-report or social desirability bias across both Health4Life and control groups (leading to lower reported alcohol and tobacco use). However, the intervention may have increased student knowledge and attitudes towards the importance of reporting alcohol and tobacco use that might not have been reported, or neglected to be reported, by those in the control group.

The results provided here differ slightly from those previously published in the primary outcomes analysis that provided little evidence for any significant change over time associated with the Health4Life intervention on all six outcomes relative to the control group (Champion et al., 2023). Some potential reasons for these differences may include the different approaches in the statistical analyses used across this and the previous study, e.g., causal mediation of effects at 24-month follow-up versus growth modelling across all time points. Importantly, O’Rourke and MacKinnon (2018) suggest that when the mediated effect and the total effect are equal in a sample, the test to determine if the mediated effects are statistically significant demonstrates substantially increased power than the test to detect a significant total effect (in some cases, the power to detect mediated effects is 11 times larger than the power to detect total effects). This can be particularly evident with large sample sizes, small effects, and when the mediators are more closely related or measured closer in time to the outcome than the intervention. As such, it might be more appropriate to focus on the effect sizes and confidence intervals across the two studies rather than the significance of the statistical tests. In this respect, both studies indicate small effects associated with all outcomes with differences between groups in the incidence of risk behaviours ranging from 2 to 3 percentage points, underscoring the importance of considering the practical significance of these effects in the context of our intervention. Moreover, the small effect sizes and the E-value analysis provide some indication that these results are at risk of being explained away by relatively weak associations with unmeasured confounders, and therefore we encourage some caution against overinterpreting these results until further research can rule out confounding and demonstrate more robust results.

There are several limitations associated with the current study that required additional discussion. First, all the outcomes were measured using student self-report, and therefore the results may have been influenced by various self-report and social desirability biases (as explained previously). Whilst it becomes prohibitively expensive and infeasible to obtain objective health data in such a large sample of school-age children, the results could benefit from additional validation under different modalities of data collection. Second, the timing of the intervention was influenced by the global COVID-19 pandemic with several lockdowns and school shutdowns occurring in the months after the intervention was administered. This once-in-a-lifetime event may have altered student behaviour to a degree that prevented any intervention effect or altered the normal response to the intervention among a large proportion of the students that might have been observed during non-pandemic times. In any case, the findings of the current study should be replicated in future samples to quantify the potential impact of the pandemic. Moreover, the current study represents one of the largest school-based cluster randomised controlled trials conducted in Australia and spans three states with each state experiencing differences with respect to the lockdowns and school closures during the pandemic. Whilst the sample does include a diverse range of schools including those from regional and socioeconomically disadvantaged areas, the sample was not recruited to be representative of the whole student population across Australia, and therefore the results may not generalise to these areas or to other Australian states. Additionally, the methods used to evaluate behavioural intentions related to sleep, screen time, SSB intake, and tobacco smoking (mediators) were specifically created for this study and have not been independently validated. This introduces the possibility of biases in measurement and reporting that could impact the results. However, these measures were adapted from the externally developed measures used to assess alcohol consumption and physical activity intentions (Newton et al., 2012; Sutherland et al., 2016). Finally, the assessment of self-efficacy focused on general self-efficacy rather than self-efficacy specific to each behaviour (e.g., self-efficacy to improve sleep). In future studies, it would be beneficial to include behaviour-specific self-efficacy measures to gain a deeper understanding of the key mechanisms that influence the effects of interventions on each risk behaviour.

This is the first study to investigate knowledge, behavioural intentions, self-efficacy, and self-control as potential mediators of an eHealth MHBC intervention administered to Australian school children. The robust causal mediation analysis provides mixed results, with small and sometimes non-significant indirect and direct effects across different outcomes rather than a consistent message that could be applied to all risk behaviours. The sensitivity analysis provided some indication that the results presented here might be subject to unmeasured confounders, and therefore caution should be taken in interpreting these results until they can be replicated in future studies.

### Supplementary Information

Below is the link to the electronic supplementary material.

Supplementary file1 (DOCX 106 KB)
