# Supplementary material for: Potential Mediators of a School-Based Digital Intervention Targeting Six Lifestyle Risk Behaviours in a Cluster Randomised Controlled Trial of Australian Adolescents
Source: Prev Sci. 2023 Dec 20;25(2):347–57. doi: 10.1007/s11121-023-01616-z (PMC10891250; doi:10.1007/s11121-023-01616-z)
Supplement: Supplementary file 1 — Supplementary file1 (DOCX 106 KB) [file 11121_2023_1616_MOESM1_ESM.docx]

**Supplementary Table 1: Mediation model estimates for alcohol use averaged across five imputed datasets.**

| Knowledge at 12 months | | Estimate | SE | Est./SE | P-value |
| --- | --- | --- | --- | --- | --- |
|  | H4L intervention | 0.610 | 0.203 | 3.004 | 0.003 |
|  | Female | 0.541 | 0.172 | 3.152 | 0.002 |
|  | Prefer not to say | 0.499 | 0.569 | 0.877 | 0.380 |
|  | Age | 0.022 | 0.090 | 0.248 | 0.804 |
|  | Psychological Distress | -0.024 | 0.014 | -1.709 | 0.087 |
|  | Alcohol intentions at baseline | -0.016 | 0.042 | -0.368 | 0.713 |
|  | Knowledge at baseline | 0.453 | 0.019 | 24.104 | 0.000 |
|  | Self-efficacy at baseline | 0.060 | 0.012 | 5.025 | 0.000 |
|  | Self-control at baseline | 0.024 | 0.010 | 2.318 | 0.020 |
|  | Alcohol use at baseline | -0.524 | 0.407 | -1.286 | 0.199 |
|  | NSW - regional | 0.272 | 0.296 | 0.918 | 0.358 |
|  | Queensland | 0.382 | 0.277 | 1.376 | 0.169 |
|  | Western Australia | 0.508 | 0.257 | 1.975 | 0.048 |
| Alcohol intentions at 12 months | |  |  |  |  |
|  | H4L intervention | -0.100 | 0.061 | -1.630 | 0.103 |
|  | Female | 0.027 | 0.053 | 0.509 | 0.611 |
|  | Prefer not to say | -0.051 | 0.173 | -0.296 | 0.767 |
|  | Age | -0.018 | 0.046 | -0.379 | 0.705 |
|  | Psychological Distress | -0.004 | 0.004 | -1.016 | 0.310 |
|  | Alcohol intentions at baseline | 0.509 | 0.015 | 33.041 | 0.000 |
|  | Knowledge at baseline | 0.018 | 0.007 | 2.452 | 0.014 |
|  | Self-efficacy at baseline | 0.010 | 0.004 | 2.812 | 0.005 |
|  | Self-control at baseline | -0.014 | 0.003 | -4.373 | 0.000 |
|  | Alcohol use at baseline | 0.377 | 0.167 | 2.264 | 0.024 |
|  | NSW - regional | 0.134 | 0.076 | 1.765 | 0.078 |
|  | Queensland | 0.122 | 0.090 | 1.360 | 0.174 |
|  | Western Australia | 0.107 | 0.080 | 1.336 | 0.182 |
| Self-efficacy at 12 months | |  |  |  |  |
|  | H4L intervention | -0.140 | 0.235 | -0.594 | 0.553 |
|  | Female | -0.435 | 0.284 | -1.531 | 0.126 |
|  | Prefer not to say | 0.831 | 0.839 | 0.991 | 0.322 |
|  | Age | 0.278 | 0.257 | 1.084 | 0.278 |
|  | Psychological Distress | -0.085 | 0.024 | -3.577 | 0.000 |
|  | Alcohol intentions at baseline | 0.077 | 0.088 | 0.870 | 0.385 |
|  | Knowledge at baseline | 0.117 | 0.043 | 2.716 | 0.007 |
|  | Self-efficacy at baseline | 0.365 | 0.022 | 16.405 | 0.000 |
|  | Self-control at baseline | 0.134 | 0.017 | 7.731 | 0.000 |
|  | Alcohol use at baseline | -0.605 | 0.661 | -0.915 | 0.360 |
|  | NSW - regional | 0.083 | 0.276 | 0.300 | 0.764 |
|  | Queensland | 0.006 | 0.303 | 0.019 | 0.985 |
|  | Western Australia | -0.372 | 0.354 | -1.052 | 0.293 |
| Self-control at 12 months | |  |  |  |  |
|  | H4L intervention | 0.035 | 0.355 | 0.100 | 0.920 |
|  | Female | -1.288 | 0.352 | -3.663 | 0.000 |
|  | Prefer not to say | -0.285 | 1.216 | -0.235 | 0.814 |
|  | Age | 0.415 | 0.294 | 1.409 | 0.159 |
|  | Psychological Distress | -0.152 | 0.026 | -5.859 | 0.000 |
|  | Alcohol intentions at baseline | -0.438 | 0.126 | -3.482 | 0.000 |
|  | Knowledge at baseline | 0.072 | 0.052 | 1.373 | 0.170 |
|  | Self-efficacy at baseline | 0.087 | 0.029 | 2.983 | 0.003 |
|  | Self-control at baseline | 0.571 | 0.020 | 28.913 | 0.000 |
|  | Alcohol use at baseline | -0.727 | 0.918 | -0.792 | 0.428 |
|  | NSW - regional | -0.231 | 0.425 | -0.545 | 0.586 |
|  | Queensland | -0.039 | 0.373 | -0.104 | 0.917 |
|  | Western Australia | -0.374 | 0.511 | -0.732 | 0.464 |
| Alcohol use at 24 months | |  |  |  |  |
|  | H4L intervention | 0.104 | 0.078 | 1.332 | 0.183 |
|  | Knowledge at 12 months | -0.028 | 0.012 | -2.431 | 0.015 |
|  | Alcohol intentions at 12 months | 0.367 | 0.041 | 8.886 | 0.000 |
|  | Self-efficacy at 12 months | 0.000 | 0.006 | -0.012 | 0.991 |
|  | Self-control at 12 months | -0.029 | 0.007 | -3.912 | 0.000 |
|  | Female | 0.062 | 0.089 | 0.696 | 0.486 |
|  | Prefer not to say | 0.401 | 0.251 | 1.594 | 0.111 |
|  | Age | 0.024 | 0.080 | 0.302 | 0.763 |
|  | Psychological distress | -0.003 | 0.008 | -0.423 | 0.672 |
|  | Alcohol intentions at baseline | 0.023 | 0.034 | 0.685 | 0.493 |
|  | Knowledge at baseline | 0.001 | 0.012 | 0.045 | 0.964 |
|  | Self-efficacy at baseline | 0.009 | 0.011 | 0.809 | 0.419 |
|  | Self-control at baseline | -0.005 | 0.005 | -0.905 | 0.365 |
|  | Alcohol use at baseline | 0.599 | 0.195 | 3.066 | 0.002 |
|  | NSW - regional | 0.164 | 0.122 | 1.345 | 0.179 |
|  | Queensland | 0.124 | 0.106 | 1.165 | 0.244 |
|  | Western Australia | -0.181 | 0.132 | -1.371 | 0.170 |

Notes: Male treated as reference category for sex at birth. NSW-metro treated as reference category for location.

**Supplementary Table 2: Mediation model estimates for moderate to vigorous physical activity averaged across five imputed datasets.**

| Knowledge at 12 months | | Estimate | SE | Est./SE | P-value |
| --- | --- | --- | --- | --- | --- |
|  | H4L intervention | 0.572 | 0.217 | 2.639 | 0.008 |
|  | Female | 0.501 | 0.181 | 2.772 | 0.006 |
|  | Prefer not to say | -0.416 | 0.437 | -0.953 | 0.341 |
|  | Age | -0.078 | 0.144 | -0.540 | 0.590 |
|  | Psychological Distress | -0.014 | 0.013 | -1.039 | 0.299 |
|  | MVPA intentions at baseline | 0.004 | 0.067 | 0.059 | 0.953 |
|  | Knowledge at baseline | 0.433 | 0.035 | 12.493 | 0.000 |
|  | Self-efficacy at baseline | 0.068 | 0.011 | 6.308 | 0.000 |
|  | Self-control at baseline | 0.024 | 0.011 | 2.189 | 0.029 |
|  | MVPA at baseline | 0.186 | 0.163 | 1.143 | 0.253 |
|  | NSW - regional | 0.357 | 0.320 | 1.116 | 0.264 |
|  | Queensland | 0.278 | 0.272 | 1.022 | 0.307 |
|  | Western Australia | 0.334 | 0.262 | 1.274 | 0.203 |
| MVPA intentions at 12 months | |  |  |  |  |
|  | H4L intervention | -0.025 | 0.064 | -0.389 | 0.697 |
|  | Female | 0.132 | 0.067 | 1.981 | 0.048 |
|  | Prefer not to say | 0.312 | 0.191 | 1.632 | 0.103 |
|  | Age | 0.034 | 0.051 | 0.674 | 0.500 |
|  | Psychological Distress | -0.011 | 0.005 | -2.213 | 0.027 |
|  | MVPA intentions at baseline | 0.499 | 0.032 | 15.709 | 0.000 |
|  | Knowledge at baseline | 0.029 | 0.008 | 3.812 | 0.000 |
|  | Self-efficacy at baseline | 0.016 | 0.006 | 2.862 | 0.004 |
|  | Self-control at baseline | 0.015 | 0.004 | 3.662 | 0.000 |
|  | MVPA at baseline | -0.388 | 0.062 | -6.308 | 0.000 |
|  | NSW - regional | -0.012 | 0.108 | -0.107 | 0.915 |
|  | Queensland | 0.013 | 0.066 | 0.204 | 0.839 |
|  | Western Australia | -0.030 | 0.120 | -0.251 | 0.802 |
| Self-efficacy at 12 months | |  |  |  |  |
|  | H4L intervention | -0.139 | 0.263 | -0.530 | 0.596 |
|  | Female | -0.447 | 0.232 | -1.928 | 0.054 |
|  | Prefer not to say | 0.228 | 1.418 | 0.161 | 0.872 |
|  | Age | 0.221 | 0.311 | 0.710 | 0.477 |
|  | Psychological Distress | -0.056 | 0.025 | -2.226 | 0.026 |
|  | MVPA intentions at baseline | 0.257 | 0.128 | 2.011 | 0.044 |
|  | Knowledge at baseline | 0.148 | 0.043 | 3.427 | 0.001 |
|  | Self-efficacy at baseline | 0.379 | 0.020 | 18.641 | 0.000 |
|  | Self-control at baseline | 0.105 | 0.027 | 3.882 | 0.000 |
|  | MVPA at baseline | -0.171 | 0.324 | -0.528 | 0.598 |
|  | NSW - regional | -0.190 | 0.385 | -0.492 | 0.622 |
|  | Queensland | -0.346 | 0.348 | -0.994 | 0.320 |
|  | Western Australia | -0.749 | 0.303 | -2.473 | 0.013 |
| Self-control at 12 months | |  |  |  |  |
|  | H4L intervention | 0.368 | 0.303 | 1.212 | 0.225 |
|  | Female | -1.148 | 0.284 | -4.036 | 0.000 |
|  | Prefer not to say | -1.023 | 1.428 | -0.716 | 0.474 |
|  | Age | 0.615 | 0.386 | 1.592 | 0.111 |
|  | Psychological Distress | -0.172 | 0.032 | -5.368 | 0.000 |
|  | MVPA intentions at baseline | -0.008 | 0.242 | -0.031 | 0.975 |
|  | Knowledge at baseline | 0.043 | 0.056 | 0.766 | 0.443 |
|  | Self-efficacy at baseline | 0.045 | 0.029 | 1.554 | 0.120 |
|  | Self-control at baseline | 0.595 | 0.022 | 27.270 | 0.000 |
|  | MVPA at baseline | -0.386 | 0.340 | -1.134 | 0.257 |
|  | NSW - regional | -0.604 | 0.476 | -1.268 | 0.205 |
|  | Queensland | -0.320 | 0.448 | -0.715 | 0.475 |
|  | Western Australia | -0.044 | 0.599 | -0.073 | 0.942 |
| MVPA at 24 months | |  |  |  |  |
|  | H4L intervention | -0.120 | 0.072 | -1.661 | 0.097 |
|  | Knowledge at 12 months | 0.015 | 0.010 | 1.526 | 0.127 |
|  | MVPA intentions at 12 months | -0.320 | 0.051 | -6.326 | 0.000 |
|  | Self-efficacy at 12 months | 0.001 | 0.005 | 0.275 | 0.783 |
|  | Self-control at 12 months | -0.008 | 0.005 | -1.630 | 0.103 |
|  | Female | 0.447 | 0.073 | 6.109 | 0.000 |
|  | Prefer not to say | -0.351 | 0.243 | -1.441 | 0.149 |
|  | Age | -0.007 | 0.066 | -0.108 | 0.914 |
|  | Psychological distress | 0.011 | 0.008 | 1.239 | 0.215 |
|  | MVPA intentions at baseline | -0.167 | 0.060 | -2.804 | 0.005 |
|  | Knowledge at baseline | 0.008 | 0.011 | 0.713 | 0.476 |
|  | Self-efficacy at baseline | 0.002 | 0.007 | 0.345 | 0.730 |
|  | Self-control at baseline | 0.006 | 0.005 | 1.253 | 0.210 |
|  | MVPA at baseline | 0.462 | 0.080 | 5.775 | 0.000 |
|  | NSW - regional | -0.185 | 0.102 | -1.824 | 0.068 |
|  | Queensland | -0.171 | 0.123 | -1.392 | 0.164 |
|  | Western Australia | 0.012 | 0.126 | 0.095 | 0.924 |

Notes: Male treated as reference category for sex at birth. NSW-metro treated as reference category for location. MVPA = moderate to vigorous physical activity.

**Supplementary Table 3: Mediation model estimates for Screen time averaged across five imputed datasets.**

| Knowledge at 12 months | | Estimate | SE | Est./SE | P-value |
| --- | --- | --- | --- | --- | --- |
|  | H4L intervention | 0.630 | 0.222 | 2.835 | 0.005 |
|  | Female | 0.540 | 0.182 | 2.971 | 0.003 |
|  | Prefer not to say | -0.350 | 0.512 | -0.684 | 0.494 |
|  | Age | 0.017 | 0.113 | 0.155 | 0.877 |
|  | Psychological Distress | -0.011 | 0.014 | -0.798 | 0.425 |
|  | Screen time intentions at baseline | 0.106 | 0.063 | 1.672 | 0.094 |
|  | Knowledge at baseline | 0.416 | 0.023 | 17.895 | 0.000 |
|  | Self-efficacy at baseline | 0.059 | 0.011 | 5.177 | 0.000 |
|  | Self-control at baseline | 0.020 | 0.009 | 2.304 | 0.021 |
|  | Screen time at baseline | -0.362 | 0.239 | -1.516 | 0.130 |
|  | NSW - regional | 0.406 | 0.361 | 1.122 | 0.262 |
|  | Queensland | 0.320 | 0.270 | 1.186 | 0.236 |
|  | Western Australia | 0.341 | 0.268 | 1.269 | 0.204 |
| Screen time intentions at 12 months | |  |  |  |  |
|  | H4L intervention | 0.087 | 0.047 | 1.848 | 0.065 |
|  | Female | 0.259 | 0.046 | 5.587 | 0.000 |
|  | Prefer not to say | 0.326 | 0.193 | 1.683 | 0.092 |
|  | Age | 0.063 | 0.041 | 1.542 | 0.123 |
|  | Psychological Distress | -0.010 | 0.004 | -2.172 | 0.030 |
|  | Screen time intentions at baseline | 0.404 | 0.025 | 16.409 | 0.000 |
|  | Knowledge at baseline | 0.016 | 0.007 | 2.388 | 0.017 |
|  | Self-efficacy at baseline | 0.011 | 0.006 | 1.996 | 0.046 |
|  | Self-control at baseline | 0.018 | 0.003 | 5.816 | 0.000 |
|  | Screen time at baseline | -0.223 | 0.076 | -2.925 | 0.003 |
|  | NSW - regional | -0.055 | 0.062 | -0.888 | 0.374 |
|  | Queensland | -0.061 | 0.070 | -0.868 | 0.385 |
|  | Western Australia | -0.083 | 0.064 | -1.299 | 0.194 |
| Self-efficacy at 12 months | |  |  |  |  |
|  | H4L intervention | -0.171 | 0.245 | -0.700 | 0.484 |
|  | Female | -0.445 | 0.252 | -1.770 | 0.077 |
|  | Prefer not to say | -0.027 | 1.233 | -0.022 | 0.982 |
|  | Age | 0.094 | 0.204 | 0.462 | 0.644 |
|  | Psychological Distress | -0.033 | 0.019 | -1.737 | 0.082 |
|  | Screen time intentions at baseline | 0.053 | 0.112 | 0.469 | 0.639 |
|  | Knowledge at baseline | 0.128 | 0.042 | 3.034 | 0.002 |
|  | Self-efficacy at baseline | 0.389 | 0.019 | 20.964 | 0.000 |
|  | Self-control at baseline | 0.108 | 0.016 | 6.867 | 0.000 |
|  | Screen time at baseline | -0.250 | 0.366 | -0.684 | 0.494 |
|  | NSW - regional | -0.274 | 0.416 | -0.658 | 0.510 |
|  | Queensland | -0.358 | 0.303 | -1.184 | 0.237 |
|  | Western Australia | -0.722 | 0.315 | -2.292 | 0.022 |
| Self-control at 12 months | |  |  |  |  |
|  | H4L intervention | 0.197 | 0.368 | 0.535 | 0.593 |
|  | Female | -1.373 | 0.298 | -4.603 | 0.000 |
|  | Prefer not to say | -1.039 | 1.381 | -0.752 | 0.452 |
|  | Age | 0.577 | 0.280 | 2.063 | 0.039 |
|  | Psychological Distress | -0.151 | 0.033 | -4.560 | 0.000 |
|  | Screen time intentions at baseline | 0.469 | 0.205 | 2.287 | 0.022 |
|  | Knowledge at baseline | 0.032 | 0.051 | 0.628 | 0.530 |
|  | Self-efficacy at baseline | 0.021 | 0.037 | 0.570 | 0.568 |
|  | Self-control at baseline | 0.585 | 0.024 | 24.102 | 0.000 |
|  | Screen time at baseline | -1.252 | 0.508 | -2.463 | 0.014 |
|  | NSW - regional | -0.638 | 0.376 | -1.697 | 0.090 |
|  | Queensland | -0.214 | 0.394 | -0.543 | 0.587 |
|  | Western Australia | -0.058 | 0.458 | -0.128 | 0.898 |
| Screen time at 24 months | |  |  |  |  |
|  | H4L intervention | 0.728 | 12.642 | 0.058 | 0.954 |
|  | Knowledge at 12 months | -0.639 | 14.410 | -0.044 | 0.965 |
|  | Screen time intentions at 12 months | -9.613 | 65.112 | -0.148 | 0.883 |
|  | Self-efficacy at 12 months | -0.866 | 5.986 | -0.145 | 0.885 |
|  | Self-control at 12 months | 1.210 | 8.820 | 0.137 | 0.891 |
|  | Female | 5.124 | 38.078 | 0.135 | 0.893 |
|  | Prefer not to say | 3.781 | 25.494 | 0.148 | 0.882 |
|  | Age | -1.631 | 11.098 | -0.147 | 0.883 |
|  | Psychological distress | -0.031 | 0.270 | -0.113 | 0.910 |
|  | Screen time intentions at baseline | 1.392 | 9.574 | 0.145 | 0.884 |
|  | Knowledge at baseline | 0.460 | 6.790 | 0.068 | 0.946 |
|  | Self-efficacy at baseline | 0.470 | 3.477 | 0.135 | 0.892 |
|  | Self-control at baseline | -0.604 | 4.420 | -0.137 | 0.891 |
|  | Screen time at baseline | 9.037 | 60.883 | 0.148 | 0.882 |
|  | NSW - regional | 0.406 | 6.488 | 0.063 | 0.950 |
|  | Queensland | -2.998 | 19.695 | -0.152 | 0.879 |
|  | Western Australia | -3.552 | 23.234 | -0.153 | 0.878 |

Notes: Male treated as reference category for sex at birth. NSW-metro treated as reference category for location.

**Supplementary Table 4: Mediation model estimates for sleep averaged across five imputed datasets.**

| Knowledge at 12 months | | Estimate | SE | Est./SE | P-value |
| --- | --- | --- | --- | --- | --- |
|  | H4L intervention | 0.618 | 0.205 | 3.009 | 0.003 |
|  | Female | 0.567 | 0.178 | 3.188 | 0.001 |
|  | Prefer not to say | -0.352 | 0.510 | -0.691 | 0.490 |
|  | Age | -0.026 | 0.108 | -0.241 | 0.809 |
|  | Psychological Distress | -0.005 | 0.012 | -0.399 | 0.690 |
|  | Sleep intentions at baseline | 0.092 | 0.056 | 1.643 | 0.100 |
|  | Knowledge at baseline | 0.410 | 0.026 | 15.956 | 0.000 |
|  | Self-efficacy at baseline | 0.068 | 0.012 | 5.511 | 0.000 |
|  | Self-control at baseline | 0.021 | 0.010 | 2.033 | 0.042 |
|  | Sleep at baseline | -0.505 | 0.135 | -3.750 | 0.000 |
|  | NSW - regional | 0.368 | 0.326 | 1.131 | 0.258 |
|  | Queensland | 0.339 | 0.273 | 1.242 | 0.214 |
|  | Western Australia | 0.305 | 0.286 | 1.065 | 0.287 |
| Sleep intentions at 12 months | |  |  |  |  |
|  | H4L intervention | 0.115 | 0.056 | 2.065 | 0.039 |
|  | Female | 0.087 | 0.044 | 2.005 | 0.045 |
|  | Prefer not to say | -0.056 | 0.197 | -0.284 | 0.777 |
|  | Age | 0.077 | 0.040 | 1.952 | 0.051 |
|  | Psychological Distress | -0.010 | 0.005 | -2.150 | 0.032 |
|  | Sleep intentions at baseline | 0.255 | 0.023 | 11.233 | 0.000 |
|  | Knowledge at baseline | 0.032 | 0.008 | 3.890 | 0.000 |
|  | Self-efficacy at baseline | 0.018 | 0.004 | 4.731 | 0.000 |
|  | Self-control at baseline | 0.016 | 0.003 | 4.718 | 0.000 |
|  | Sleep at baseline | -0.215 | 0.042 | -5.147 | 0.000 |
|  | NSW - regional | -0.084 | 0.077 | -1.084 | 0.278 |
|  | Queensland | 0.003 | 0.070 | 0.039 | 0.969 |
|  | Western Australia | -0.112 | 0.070 | -1.609 | 0.108 |
| Self-efficacy at 12 months | |  |  |  |  |
|  | H4L intervention | -0.167 | 0.312 | -0.535 | 0.593 |
|  | Female | -0.419 | 0.355 | -1.180 | 0.238 |
|  | Prefer not to say | -0.197 | 0.954 | -0.207 | 0.836 |
|  | Age | 0.263 | 0.230 | 1.145 | 0.252 |
|  | Psychological Distress | -0.047 | 0.027 | -1.738 | 0.082 |
|  | Sleep intentions at baseline | 0.014 | 0.133 | 0.103 | 0.918 |
|  | Knowledge at baseline | 0.110 | 0.035 | 3.130 | 0.002 |
|  | Self-efficacy at baseline | 0.387 | 0.028 | 13.672 | 0.000 |
|  | Self-control at baseline | 0.105 | 0.020 | 5.344 | 0.000 |
|  | Sleep at baseline | 0.002 | 0.228 | 0.007 | 0.995 |
|  | NSW - regional | -0.164 | 0.382 | -0.429 | 0.668 |
|  | Queensland | -0.350 | 0.367 | -0.955 | 0.340 |
|  | Western Australia | -0.636 | 0.299 | -2.129 | 0.033 |
| Self-control at 12 months | |  |  |  |  |
|  | H4L intervention | 0.136 | 0.326 | 0.417 | 0.677 |
|  | Female | -1.129 | 0.287 | -3.937 | 0.000 |
|  | Prefer not to say | -0.443 | 1.619 | -0.273 | 0.785 |
|  | Age | 0.727 | 0.265 | 2.742 | 0.006 |
|  | Psychological Distress | -0.149 | 0.034 | -4.349 | 0.000 |
|  | Sleep intentions at baseline | 0.119 | 0.148 | 0.801 | 0.423 |
|  | Knowledge at baseline | 0.058 | 0.054 | 1.074 | 0.283 |
|  | Self-efficacy at baseline | 0.040 | 0.029 | 1.372 | 0.170 |
|  | Self-control at baseline | 0.596 | 0.021 | 28.779 | 0.000 |
|  | Sleep at baseline | -0.350 | 0.308 | -1.136 | 0.256 |
|  | NSW - regional | -0.826 | 0.461 | -1.791 | 0.073 |
|  | Queensland | -0.170 | 0.401 | -0.425 | 0.671 |
|  | Western Australia | -0.058 | 0.452 | -0.128 | 0.898 |
| Sleep at 24 months | |  |  |  |  |
|  | H4L intervention | -0.076 | 0.066 | -1.156 | 0.248 |
|  | Knowledge at 12 months | -0.011 | 0.009 | -1.270 | 0.204 |
|  | Sleep intentions at 12 months | -0.161 | 0.037 | -4.355 | 0.000 |
|  | Self-efficacy at 12 months | -0.006 | 0.004 | -1.448 | 0.148 |
|  | Self-control at 12 months | -0.006 | 0.004 | -1.482 | 0.138 |
|  | Female | -0.096 | 0.070 | -1.371 | 0.170 |
|  | Prefer not to say | 0.185 | 0.257 | 0.719 | 0.472 |
|  | Age | 0.071 | 0.056 | 1.270 | 0.204 |
|  | Psychological distress | 0.016 | 0.005 | 2.954 | 0.003 |
|  | Sleep intentions at baseline | -0.049 | 0.029 | -1.678 | 0.093 |
|  | Knowledge at baseline | -0.016 | 0.011 | -1.477 | 0.140 |
|  | Self-efficacy at baseline | 0.011 | 0.006 | 1.744 | 0.081 |
|  | Self-control at baseline | 0.003 | 0.004 | 0.670 | 0.503 |
|  | Sleep at baseline | 0.393 | 0.058 | 6.827 | 0.000 |
|  | NSW - regional | 0.028 | 0.090 | 0.309 | 0.757 |
|  | Queensland | 0.030 | 0.080 | 0.374 | 0.708 |
|  | Western Australia | -0.136 | 0.067 | -2.018 | 0.044 |

Notes: Male treated as reference category for sex at birth. NSW-metro treated as reference category for location.

**Supplementary Table 5: Mediation model estimates for sugar-sweetened beverages averaged across five imputed datasets.**

| Knowledge at 12 months | | Estimate | SE | Est./SE | P-value |
| --- | --- | --- | --- | --- | --- |
|  | H4L intervention | 0.634 | 0.207 | 3.064 | 0.002 |
|  | Female | 0.422 | 0.177 | 2.381 | 0.017 |
|  | Prefer not to say | -0.435 | 0.415 | -1.050 | 0.294 |
|  | Age | -0.022 | 0.119 | -0.186 | 0.852 |
|  | Psychological Distress | -0.007 | 0.014 | -0.488 | 0.626 |
|  | SSB intentions at baseline | 0.071 | 0.053 | 1.351 | 0.177 |
|  | Knowledge at baseline | 0.412 | 0.023 | 17.742 | 0.000 |
|  | Self-efficacy at baseline | 0.065 | 0.012 | 5.667 | 0.000 |
|  | Self-control at baseline | 0.022 | 0.010 | 2.229 | 0.026 |
|  | SSB at baseline | -0.628 | 0.146 | -4.302 | 0.000 |
|  | NSW - regional | 0.366 | 0.340 | 1.079 | 0.281 |
|  | Queensland | 0.269 | 0.270 | 0.995 | 0.320 |
|  | Western Australia | 0.230 | 0.268 | 0.858 | 0.391 |
| SSB intentions at 12 months | |  |  |  |  |
|  | H4L intervention | 0.039 | 0.059 | 0.665 | 0.506 |
|  | Female | 0.102 | 0.046 | 2.209 | 0.027 |
|  | Prefer not to say | 0.150 | 0.176 | 0.854 | 0.393 |
|  | Age | 0.012 | 0.041 | 0.295 | 0.768 |
|  | Psychological Distress | 0.000 | 0.005 | -0.099 | 0.921 |
|  | SSB intentions at baseline | 0.227 | 0.018 | 12.808 | 0.000 |
|  | Knowledge at baseline | 0.022 | 0.008 | 2.761 | 0.006 |
|  | Self-efficacy at baseline | 0.013 | 0.004 | 3.408 | 0.001 |
|  | Self-control at baseline | 0.009 | 0.004 | 2.424 | 0.015 |
|  | SSB at baseline | -0.199 | 0.053 | -3.750 | 0.000 |
|  | NSW - regional | -0.064 | 0.064 | -0.989 | 0.322 |
|  | Queensland | -0.071 | 0.057 | -1.235 | 0.217 |
|  | Western Australia | 0.014 | 0.083 | 0.168 | 0.866 |
| Self-efficacy at 12 months | |  |  |  |  |
|  | H4L intervention | -0.120 | 0.244 | -0.492 | 0.622 |
|  | Female | -0.579 | 0.251 | -2.303 | 0.021 |
|  | Prefer not to say | 0.001 | 1.068 | 0.001 | 0.999 |
|  | Age | 0.241 | 0.230 | 1.048 | 0.295 |
|  | Psychological Distress | -0.043 | 0.024 | -1.763 | 0.078 |
|  | SSB intentions at baseline | -0.014 | 0.097 | -0.147 | 0.883 |
|  | Knowledge at baseline | 0.134 | 0.038 | 3.538 | 0.000 |
|  | Self-efficacy at baseline | 0.385 | 0.021 | 18.388 | 0.000 |
|  | Self-control at baseline | 0.110 | 0.016 | 7.052 | 0.000 |
|  | SSB at baseline | -0.251 | 0.252 | -0.997 | 0.319 |
|  | NSW - regional | -0.048 | 0.407 | -0.118 | 0.906 |
|  | Queensland | -0.357 | 0.327 | -1.089 | 0.276 |
|  | Western Australia | -0.699 | 0.306 | -2.285 | 0.022 |
| Self-control at 12 months | |  |  |  |  |
|  | H4L intervention | 0.137 | 0.316 | 0.434 | 0.664 |
|  | Female | -1.123 | 0.282 | -3.986 | 0.000 |
|  | Prefer not to say | -0.818 | 1.816 | -0.450 | 0.652 |
|  | Age | 0.639 | 0.299 | 2.137 | 0.033 |
|  | Psychological Distress | -0.167 | 0.029 | -5.803 | 0.000 |
|  | SSB intentions at baseline | 0.006 | 0.141 | 0.040 | 0.968 |
|  | Knowledge at baseline | 0.044 | 0.047 | 0.924 | 0.355 |
|  | Self-efficacy at baseline | 0.023 | 0.031 | 0.751 | 0.453 |
|  | Self-control at baseline | 0.611 | 0.024 | 25.600 | 0.000 |
|  | SSB at baseline | -0.149 | 0.402 | -0.371 | 0.711 |
|  | NSW - regional | -0.689 | 0.353 | -1.953 | 0.051 |
|  | Queensland | -0.368 | 0.355 | -1.036 | 0.300 |
|  | Western Australia | -0.099 | 0.383 | -0.258 | 0.796 |
| SSB at 24 months | |  |  |  |  |
|  | H4L intervention | 0.065 | 0.049 | 1.316 | 0.188 |
|  | Knowledge at 12 months | -0.017 | 0.011 | -1.587 | 0.113 |
|  | SSB intentions at 12 months | -0.099 | 0.038 | -2.591 | 0.010 |
|  | Self-efficacy at 12 months | 0.003 | 0.006 | 0.471 | 0.638 |
|  | Self-control at 12 months | -0.009 | 0.004 | -2.054 | 0.040 |
|  | Female | -0.393 | 0.061 | -6.467 | 0.000 |
|  | Prefer not to say | 0.148 | 0.245 | 0.602 | 0.547 |
|  | Age | 0.029 | 0.057 | 0.509 | 0.611 |
|  | Psychological distress | -0.006 | 0.006 | -1.036 | 0.300 |
|  | SSB intentions at baseline | -0.033 | 0.025 | -1.337 | 0.181 |
|  | Knowledge at baseline | -0.001 | 0.012 | -0.097 | 0.923 |
|  | Self-efficacy at baseline | -0.006 | 0.006 | -1.053 | 0.292 |
|  | Self-control at baseline | -0.004 | 0.005 | -0.867 | 0.386 |
|  | SSB at baseline | 0.929 | 0.065 | 14.255 | 0.000 |
|  | NSW - regional | 0.104 | 0.076 | 1.365 | 0.172 |
|  | Queensland | 0.162 | 0.067 | 2.435 | 0.015 |
|  | Western Australia | -0.025 | 0.085 | -0.299 | 0.765 |

Notes: Male treated as reference category for sex at birth. NSW-metro treated as reference category for location. SSB = sugar-sweetened beverages.

**Supplementary Table 5: Mediation model estimates for tobacco use averaged across five imputed datasets.**

| Knowledge at 12 months | | Estimate | SE | Est./SE | P-value |
| --- | --- | --- | --- | --- | --- |
|  | H4L intervention | 0.651 | 0.209 | 3.114 | 0.002 |
|  | Female | 0.530 | 0.195 | 2.720 | 0.007 |
|  | Prefer not to say | -0.380 | 0.494 | -0.769 | 0.442 |
|  | Age | -0.023 | 0.112 | -0.201 | 0.840 |
|  | Psychological Distress | -0.012 | 0.012 | -1.017 | 0.309 |
|  | Tobacco use intentions at baseline | -0.315 | 0.087 | -3.600 | 0.000 |
|  | Knowledge at baseline | 0.421 | 0.022 | 18.867 | 0.000 |
|  | Self-efficacy at baseline | 0.058 | 0.011 | 5.403 | 0.000 |
|  | Self-control at baseline | 0.022 | 0.009 | 2.489 | 0.013 |
|  | Tobacco use at baseline | 0.152 | 0.442 | 0.343 | 0.732 |
|  | NSW - regional | 0.424 | 0.331 | 1.279 | 0.201 |
|  | Queensland | 0.306 | 0.294 | 1.039 | 0.299 |
|  | Western Australia | 0.256 | 0.263 | 0.976 | 0.329 |
| Tobacco use intentions at 12 months | |  |  |  |  |
|  | H4L intervention | -0.061 | 0.069 | -0.888 | 0.375 |
|  | Female | -0.005 | 0.063 | -0.076 | 0.940 |
|  | Prefer not to say | 0.025 | 0.262 | 0.096 | 0.924 |
|  | Age | -0.036 | 0.057 | -0.629 | 0.529 |
|  | Psychological Distress | 0.000 | 0.005 | 0.012 | 0.990 |
|  | Tobacco use intentions at baseline | 0.562 | 0.036 | 15.653 | 0.000 |
|  | Knowledge at baseline | -0.003 | 0.008 | -0.388 | 0.698 |
|  | Self-efficacy at baseline | 0.004 | 0.005 | 0.823 | 0.410 |
|  | Self-control at baseline | -0.030 | 0.004 | -7.240 | 0.000 |
|  | Tobacco use at baseline | 0.203 | 0.172 | 1.180 | 0.238 |
|  | NSW - regional | 0.074 | 0.092 | 0.803 | 0.422 |
|  | Queensland | 0.015 | 0.097 | 0.154 | 0.878 |
|  | Western Australia | -0.168 | 0.099 | -1.693 | 0.090 |
| Self-efficacy at 12 months | |  |  |  |  |
|  | H4L intervention | -0.142 | 0.326 | -0.436 | 0.663 |
|  | Female | -0.480 | 0.236 | -2.036 | 0.042 |
|  | Prefer not to say | 0.043 | 0.860 | 0.050 | 0.960 |
|  | Age | 0.198 | 0.203 | 0.975 | 0.330 |
|  | Psychological Distress | -0.049 | 0.022 | -2.242 | 0.025 |
|  | Tobacco use intentions at baseline | -0.200 | 0.132 | -1.516 | 0.129 |
|  | Knowledge at baseline | 0.151 | 0.039 | 3.919 | 0.000 |
|  | Self-efficacy at baseline | 0.391 | 0.023 | 16.998 | 0.000 |
|  | Self-control at baseline | 0.103 | 0.015 | 6.925 | 0.000 |
|  | Tobacco use at baseline | -0.043 | 0.988 | -0.043 | 0.965 |
|  | NSW - regional | -0.151 | 0.396 | -0.380 | 0.704 |
|  | Queensland | -0.231 | 0.334 | -0.692 | 0.489 |
|  | Western Australia | -0.866 | 0.321 | -2.701 | 0.007 |
| Self-control at 12 months | |  |  |  |  |
|  | H4L intervention | 0.159 | 0.281 | 0.567 | 0.571 |
|  | Female | -1.280 | 0.286 | -4.471 | 0.000 |
|  | Prefer not to say | -1.646 | 1.748 | -0.941 | 0.347 |
|  | Age | 0.769 | 0.319 | 2.409 | 0.016 |
|  | Psychological Distress | -0.158 | 0.026 | -6.183 | 0.000 |
|  | Tobacco use intentions at baseline | -0.735 | 0.240 | -3.067 | 0.002 |
|  | Knowledge at baseline | 0.048 | 0.047 | 1.030 | 0.303 |
|  | Self-efficacy at baseline | 0.037 | 0.031 | 1.171 | 0.242 |
|  | Self-control at baseline | 0.590 | 0.022 | 26.896 | 0.000 |
|  | Tobacco use at baseline | -0.425 | 1.129 | -0.377 | 0.706 |
|  | NSW - regional | -0.794 | 0.344 | -2.310 | 0.021 |
|  | Queensland | -0.158 | 0.399 | -0.395 | 0.693 |
|  | Western Australia | -0.171 | 0.461 | -0.370 | 0.711 |
| Tobacco use at 24 months | |  |  |  |  |
|  | H4L intervention | 2.172 | 16.140 | 0.135 | 0.893 |
|  | Knowledge at 12 months | -0.742 | 10.549 | -0.070 | 0.944 |
|  | Tobacco use intentions at 12 months | 10.579 | 66.048 | 0.160 | 0.873 |
|  | Self-efficacy at 12 months | 0.278 | 5.759 | 0.048 | 0.962 |
|  | Self-control at 12 months | -0.742 | 8.790 | -0.084 | 0.933 |
|  | Female | -0.170 | 9.800 | -0.017 | 0.986 |
|  | Prefer not to say | 2.734 | 43.300 | 0.063 | 0.950 |
|  | Age | 1.408 | 9.457 | 0.149 | 0.882 |
|  | Psychological distress | -0.132 | 1.516 | -0.087 | 0.931 |
|  | Tobacco use intentions at baseline | -3.705 | 21.801 | -0.170 | 0.865 |
|  | Knowledge at baseline | -0.003 | 1.593 | -0.002 | 0.998 |
|  | Self-efficacy at baseline | -0.098 | 1.187 | -0.083 | 0.934 |
|  | Self-control at baseline | 0.308 | 3.454 | 0.089 | 0.929 |
|  | Tobacco use at baseline | 1.745 | 15.911 | 0.110 | 0.913 |
|  | NSW - regional | 1.814 | 14.289 | 0.127 | 0.899 |
|  | Queensland | 1.613 | 11.704 | 0.138 | 0.890 |
|  | Western Australia | 1.677 | 11.159 | 0.150 | 0.881 |

Notes: Male treated as reference category for sex at birth. NSW-metro treated as reference category for location.
